# The efficacy of aminosalicylates in acute radiation enteritis: a systematic review and meta-analysis

**DOI:** 10.3389/fphar.2025.1544981

**Published:** 2025-04-22

**Authors:** Zhendong Wu, Chuyan Ni, Zhen Ye, Zhongsheng Xia, Li Li, Zhong Yu, Song Tang, Ying Lin, Wa Zhong

**Affiliations:** ^1^ Department of Gastroenterology, Sun Yat-Sen Memorial Hospital, Sun Yat-Sen University, Guangzhou, Guangdong, China; ^2^ Department of Gastroenterology, Dongguan Songshan Lake Tungwah Hospital, Dongguan, Guangdong, China; ^3^ Department of Gastroenterology, Longgang District People’s Hospital of Shenzhen, Shenzhen, Guangdong, China; ^4^ Guangdong Provincial Key Laboratory of Malignant Tumor Epigenetics and Gene Regulation, Sun Yat-Sen Memorial Hospital, Sun Yat-Sen University, Guangzhou, Guangdong, China; ^5^ Department of Emergency, Sun Yat-Sen Memorial Hospital, Sun Yat-Sen University, Guangzhou, Guangdong, China; ^6^ Department of Gastrointestinal Surgery, The First Affiliated Hospital of Guangdong Pharmaceutical University, Guangzhou, Guangdong, China

**Keywords:** aminosalicylates, SASP, 5-ASA, radiation enteritis, meta-analysis

## Abstract

**Background and purpose:**

Aminosalicylates have been used for the prevention and treatment of radiation enteritis (RE) for more than 50 years. However, their effectiveness in acute radiation enteritis (ARE) has been controversial. We conducted a meta-analysis to clarify the clinical efficacy of aminosalicylates in controlling the symptoms of ARE.

**Materials and methods:**

We searched PubMed, Cochrane Library, Embase, and Web of Science for studies published before January 2020. Eligible randomized controlled trials (RCTs) comparing the incidence of diarrhea, abdominal pain, constipation, tenesmus, and hematochezia between the aminosalicylates and control groups were included. Subgroup analyses were conducted based on different drugs and doses. Publication bias was assessed using funnel plots.

**Results:**

Seven RCTs with 613 patients were included. Aminosalicylates reduced the incidence of mild to moderate diarrhea (P < 0.05), while total diarrhea, severe diarrhea, abdominal pain, hematochezia, tenesmus, and constipation showed no significant differences from the control group. Subgroup analysis showed that sulfasalazine (SASP) reduced mild to moderate diarrhea (P < 0.05), whereas 5-aminosalicylic acid (5-ASA) increased total and severe diarrhea (P < 0.05). Additionally, when aminosalicylate doses exceeded 2 g/d, diarrhea incidence increased (P < 0.05).

**Conclusion:**

SASP is a safe and effective treatment for mild to moderate diarrhea, while 5-ASA may increase diarrhea incidence in ARE patients. Aminosalicylates at ≤2 g/d are safe for ARE, but higher doses may worsen diarrhea.

## Introduction

Globocan 2020 data indicates that pelvic malignancies account for 28.4% of all cancer cases globally (Sung et al., 2021). Radiation therapy is an essential treatment for pelvic malignancies, but approximately 90% of patients undergo permanent changes in bowel habits following pelvic radiation therapy (Andreyev, 2007). Acute radiation enteritis (ARE) refers to gastrointestinal toxicity occurring within 3 months after radiation therapy, involving the small intestine, colon, and rectum, and characterized by symptoms such as diarrhea, abdominal pain, constipation, tenesmus and rectal bleeding (Loge et al., 2020; P et al., 2024; Zimmerer et al., 2008). With the increase of tumor incidence, the prevalence of RE is also on the rise. It affects not only the continuity of anti-tumor therapy, but also reduces the quality of life for the patients. Even a single day of unplanned interruption during radiation therapy can potentially reduce local control rates by 1%–1.4% (P et al., 2024). However, there are no gold standard for the prevention and treatment of acute radiation enteritis.

Aminosalicylates are the essential treatment for inflammatory bowel diseases (IBDs), which have also been used for the prevention and treatment of RE for more than 50 years (Rauch and Weiland, 1972; Cai et al., 2021). Among them, sulfasalazine (SASP) and 5-aminosalicylic acid (5-ASA) are the most frequently prescribed agents for symptom control in ARE. However, the effectiveness of aminosalicylates in ARE remains controversial. In a 1993 randomized trial, Baughan et al. (1993) found that 5-ASA increased the incidence of diarrhea in the patients who received pelvic radiotherapy compared with placebo, raising concerns about its therapeutic role. Subsequent randomized controlled trials (RCTs) yielded conflicting results, with some studies finding that 5-ASA failed to alleviate symptoms of RE (Martenson et al., 1996; Resbeut et al., 1997; Sanguineti et al., 2003). Furthermore, discrepancies exist regarding the efficacy of SASP. While Miller et al. (2016) observed no significant reduction in diarrhea, abdominal pain, hematochezia, or tenesmus with SASP treatment, two other RCTs (Kiliç et al., 2000; Pal et al., 2013) reported a lower incidence of diarrhea in SASP-treated patients compared with placebo.

These inconsistencies in prior studies may be attributed to differences in study design, patient populations, radiation dosages, and drug regimens. Additionally, variations in outcome definitions and assessment criteria further complicate the interpretation of results. Given these uncertainties, a comprehensive meta-analysis is warranted to systematically evaluate the clinical efficacy of aminosalicylates (SASP and 5-ASA) in preventing and treating ARE.

## Materials and methods

### Eligibility

RCTs comparing SASP and 5-ASA with placebo or control drugs for relieving the clinical symptoms of ARE were included in this meta-analysis. The studies had to report data on clinical efficacy and/or adverse events. To ensure comprehensive coverage and convenience, only studies published in English or Chinese were included. Animal studies were excluded. Additionally, studies were excluded if they were duplicate publications, had incomplete data, contained missing data, exhibited statistical errors, or assessed efficacy without using standardized criteria.

### Information sources and search

Eligible studies were searched in Pubmed, The Cochrane Library, Embase and Web of Science for publications dated before January 2020. The systematic search was performed with the following terms “5-aminosalicylate,” “5-aminosalicylic acid,” “5-ASA,” “sulfasalazine,” “SASP,” “radiation enteritis,” “radiation proctocolitis,” “radiotherapy,” “RE,” and “enteritis”. The terms were grouped in different combinations to search across different databases. For articles without full text, we contacted the authors to obtain the study details.

### Study selection

The titles and abstracts of the identified studies were reviewed. Potentially eligible articles were collected for full-text review. In cases where there were disagreements about inclusion, discussions were held, and if necessary, a third reviewer was consulted to make the final decision.

### Data extraction and quality assessment

General data from the eligible studies were collected independently by two researchers, including the name of the first author, year of publication, study type, primary disease, radiation dose, number of participants, participants’ age and gender, form and dose of the drug, therapeutic approach and duration, as well as the incidence of total diarrhea, mild to moderate diarrhea, severe diarrhea, abdominal pain, constipation, hematochezia, and tenesmus. Adverse events and follow-up time were also recorded. If any of the aforementioned data were unavailable (NA), it was noted as NA. Since most of the included studies were randomized controlled trials, the quality was assessed based on the methods outlined in the Cochrane 5.1.0 handbook (Higgins et al., 2011), considering factors such as randomization, blinding, complete reporting, and other potential biases.

The Cochrane Risk of Bias tool is widely used for quality assessment in RCT studies. The assessment items include: generation of random sequences, allocation concealment, blinding of participants and personnel, blinding of outcome assessment, incomplete outcome data, selective reporting, and other biases. In the Cochrane quality assessment, if all items are rated as “low risk,” the likelihood of various biases is minimized, and the overall score is rated as Grade A, indicating a high-quality study. If one or more items are rated as “unclear,” there is a moderate possibility of corresponding biases, and the overall score is rated as Grade B, indicating a medium-quality study. If one or more items are rated as “high risk,” the likelihood of corresponding biases is high, and the overall score is rated as Grade C, indicating a low-quality study.

### Statistical analysis

The efficacy of aminosalicylates was evaluated according to the incidence of diarrhea, abdominal pain, constipation, hematochezia, and tenesmus. Additionally, the different forms and doses of the drug, therapeutic approach, and duration were also recorded. The relative risk (RR) was used as the combined statistic, and the Z-test was employed to obtain the probability P-value. If P < 0.05, the difference in efficacy between the two groups was considered statistically significant. If P > 0.05, the difference in efficacy was considered not statistically significant. The included study data were summarized, and subgroup analyses were conducted based on the type and dosage of ASA agents to compare the effects of different drug types and dosages on efficacy. The meta-analysis of included studies was conducted using the Review manager 5.3 software. The inconsistency value (I^2^) was calculated to assess heterogeneity between eligible trials. A random-effects model or subgroup analysis was applied if the heterogeneity was significant (P < 0.1, I^2^>50%). Otherwise, the fixed effect model was used. The Z-test was performed with relative RR to synthesize statistics across the trials. The difference was considered statistically significant while P < 0.05. Funnel plots were employed to detect potential publication bias.

## Results

### Study selection and baseline characteristics

**FIGURE 1:**
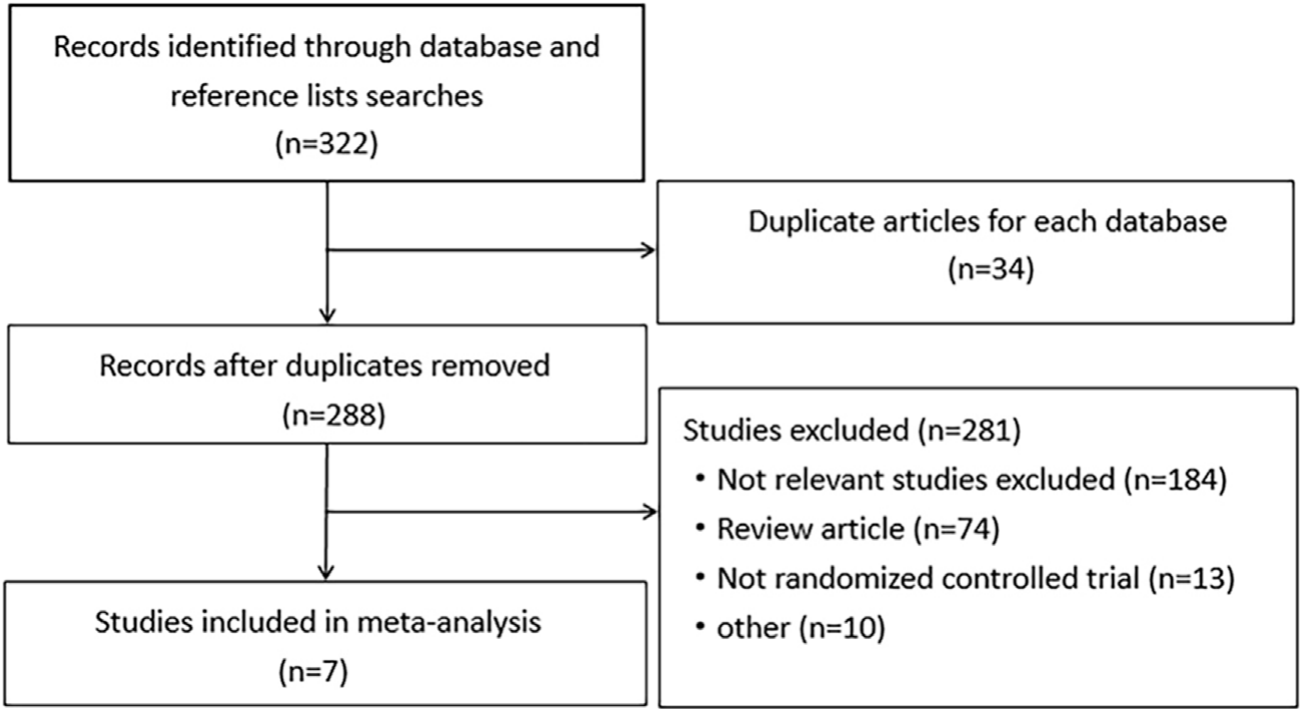
The flow chart for screening studies in the meta-analysis.

A total of 321 relevant studies were obtained from the databases with searching terms described above. Of these, 314 studies were excluded for not meeting the inclusion criteria. Seven studies were included for meta-analysis (Baughan et al., 1993; Martenson et al., 1996; Resbeut et al., 1997; Sanguineti et al., 2003; Miller et al., 2016; Kiliç et al., 2000; Pal et al., 2013). They were randomized, double-blinded controlled trials with well described inclusion and exclusion criteria. The use of drugs was described in detail. Except for an uncompleted study, other studies had comprehensive research data. The workflow of study selection was shown in Figure 1. The baseline characteristics were summarized in Table 1. A total of 613 patients were included in the 7 studies, in which 279 patients were treated with aminosalicylates, 278 with placebo and 56 with sucralfate. The age, sex and radiation dose were comparable among these groups (Table 1).

**TABLE 1:** Characteristic of Studies included in the Meta-Analysis.

No.	1st author year	Journal	Study type	Numbers of patients in Total/Group a/Group b	Radiation dosage (Gy)	Tumor types (cases)	Group a	Group b	Duration of use	Other treatments
1	Miller (2016)	Int J Radiat Oncol Biol Phys	RCT	Total:N = 84; 37–84y Group a: n = 42 Group b: n = 42	45.0–53.0 or >53.0	Colorectal cancer (51) Gynecological tumor (16) Prostate carcinoma (15) Pelvic tumor (1) Others (1)	Oral Sulfasalazine (2.0 g/d)	Placebo	During radiotherapy to 4 weeks after radiotherapy	Fluorouracil Capecitabine Oxaliplation
2	Pal (2013)	Clin Cancer Invest J	RCT	Total:N = 95; 35–70y Group a: n = 47 Group b: n = 48	50.0	Gynecological tumor (95)	Oral Sulfasalazine (2.0 g/d)	Placebo	During radiotherapy to 1 week after radiotherapy	Cisplatinum
3	Sanguineti (2003)	Strahlenther Onkol	RCT	Total:N = 64; 52–89y Group a: n = 8 Group b: n = 56	76.0	Prostate carcinoma (64)	Mesalazine enemas (4.0 g/d)	Sucralfate enemas	During radiotherapy	No
4	Kilic (2000)	Radiother Oncol	RCT	Total: N = 87 Group a: n = 44; 59y Group b: n = 43; 61y	46–50	Colorectal cancer (42) Gynecological tumor (22) Urologic neoplasms (22) Pelvic sarcoma (1)	Oral Sulfasalazine (2.0 g/d)	Placebo	During radiotherapy	No
5	Resbeut (1997)	Radiother Oncol	RCT	Total: N = 153 Group a: n = 74; 64y Group b: n = 79; 62.8y	45–52	Prostate carcinoma (99) Gynecological tumor (54)	Oral Mesalazine (4.0 g/d)	Placebo	During radiotherapy	No
6	Martenson (1996)	Int J Radiat Oncol Biol Phys	RCT	Total: N = 58 Group a: n = 30; 69y Group b: n = 28; 70y	45–53.5	Colorectal cancer (5) Gynecological tumor (9) Urologic neoplasms (44)	Oral Olsalazine (1.0 g/d)	Placebo	During radiotherapy	Fluorouracil Levamisole
7	Baughan (1993)	Clin Oncol	RCT	Total: N = 72 Group a: n = 34; N/A Group b: n = 38; N/A	30–60	Urologic neoplasms (55) Gynecological tumor (15) Colorectal cancer (2)	Oral Mesalazine (2.4 g/d)	Placebo	1 day before radiotherapy to 4 weeks after radiotherapy	No

No.	Clinical grading standards	Incidence of total diarrhea (%)	Incidence of mild-to-moderate diarrhea (%)	Incidence of severe diarrhea (%)	Rate of using antidiarrheal medications (%)	Incidence of abdominal pain (%)	Incidence of constipation (%)	Incidence of hematochezia (%)	Incidence of tenesmus (%)
1	CTCv4.0	Group a: 76.2 Group b: 76.2	Group a: 50.0 Group b: 66.7	Group a: 26.2 Group b: 9.5	Group a: 50.0 Group b: 28.6	Group a: 47.6 Group b: 38.1	Group a: 38.1 Group b: 35.7	Group a: 38.1 Group b: 47.6	Group a:35.7 Group b:28.6
2	CTCv4.0	Group a: 44.7 Group b: 75.0	Group a: 40.4 Group b: 58.3	Group a: 4.3 Group b: 16.7	N/A	N/A	N/A	N/A	N/A
3	RTOG	Group a: 87.5 Group b: 62.5	Group a: 25.0 Group b: 26.8	Group a: 62.5 Group b: 35.7	N/A	N/A	N/A	N/A	N/A
4	Lent-soma	Group a: 54.5 Group b: 86.0	Group a: 47.7 Group b: 55.8	Group a: 6.8 Group b: 30.2	N/A	N/A	N/A	N/A	N/A
5	WHO	Group a: 70.3 Group b: 65.8	Group a: 56.8 Group b: 58.2	Group a: 13.5 Group b: 7.6	N/A	Group a: 33.8 Group b: 50.6	N/A	N/A	N/A
6	CTC	Group a: 80.0 Group b: 75.0	Group a: 20.0 Group b: 60.7	Group a: 60.0 Group b: 14.3	N/A	Group a: 53.3 Group b: 32.1	N/A	Group a: 26.7 Group b: 32.1	Group a:13.3 Group b:39.3
7	N/A	Group a: 91.2 Group b: 73.7	Group a: 52.9 Group b: 60.5	Group a: 38.2 Group b: 13.2	Group a: 55.9 Group b: 26.3	N/A	Group a: 32.4 Group b: 34.2	Group a: 8.8 Group b: 5.3	N/A

Group a: aminosalicylic acid group; Group b: control group; RCT, randomized controlled trial; N/A, data not available.

### Efficacy of aminosalicylates on relieving symptoms of radiation enteritis

#### Diarrhea

**FIGURE 2:**
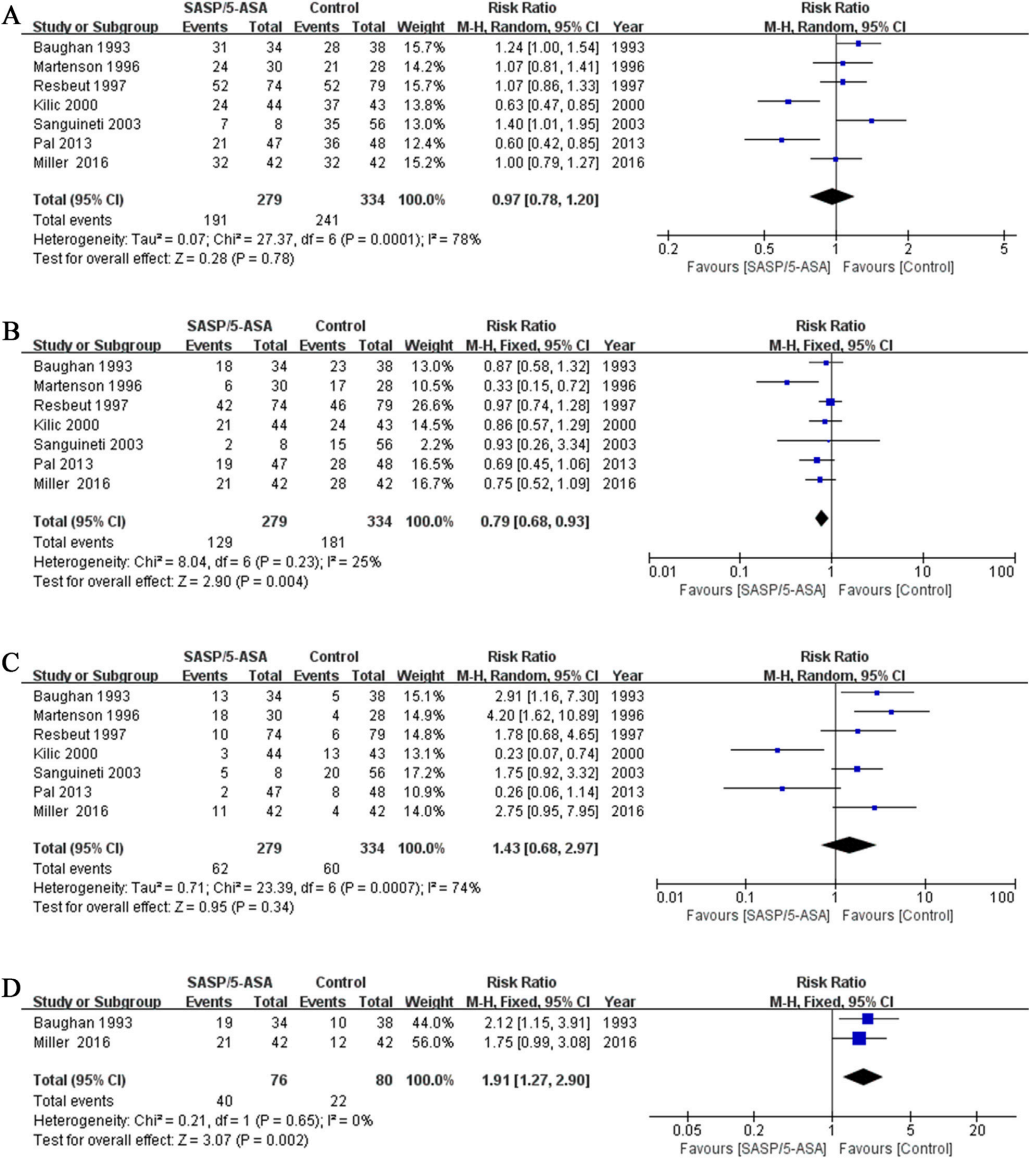
The forest plots of RR for aminosalicylate in relieving diarrhea in patients with ARE. **(A)** Total diarrhea. **(B)** Mild to moderate diarrhea. **(C)** Severe diarrhea. **(D)** Incidence of using antidiarrheal medications.

In the seven studies, the primary endpoint was the maximum severity of diarrhea for each patient. The incidence of total diarrhea did not differ significantly between the aminosalicylates and control groups (68.5% vs. 72.2%; Q = 27.37, I^2^ = 78.1%, P = 0.000; Z = 0.28, P = 0.78; Figure 2A). The incidence of mild to moderate diarrhea (grade I and II)in the aminosalicylates group was significantly lower than that in the control group (46.2% vs. 54.2%; Q = 8.04, I^2^ = 25%, P = 0.23; Z = 2.90, P = 0.004; Figure 2B). In contrast, the incidence of severe diarrhea (grade III and IV) in the aminosalicylates group was similar to that in the control group (22.2% vs. 18.0%; Q = 23.39, I^2^ = 74%, P = 0.001; Z = 0.95, P = 0.34; Figure 2C).

These results indicated that aminosalicylates could reduce the incidence of mild to moderate diarrhea in the ARE patients. However, it may not be effective in relieving severe diarrhea.

In addition, the proportion of patients using anti-diarrhea medications in the aminosalicylates group was significantly higher than that in the control group (52.6% vs. 27.5%) in two studies (Q = 0.21, I^2^ = 0%, P = 0.65; Z = 3.07, P = 0.002; Figure 2D).

#### Abdominal pain

**FIGURE 3:**
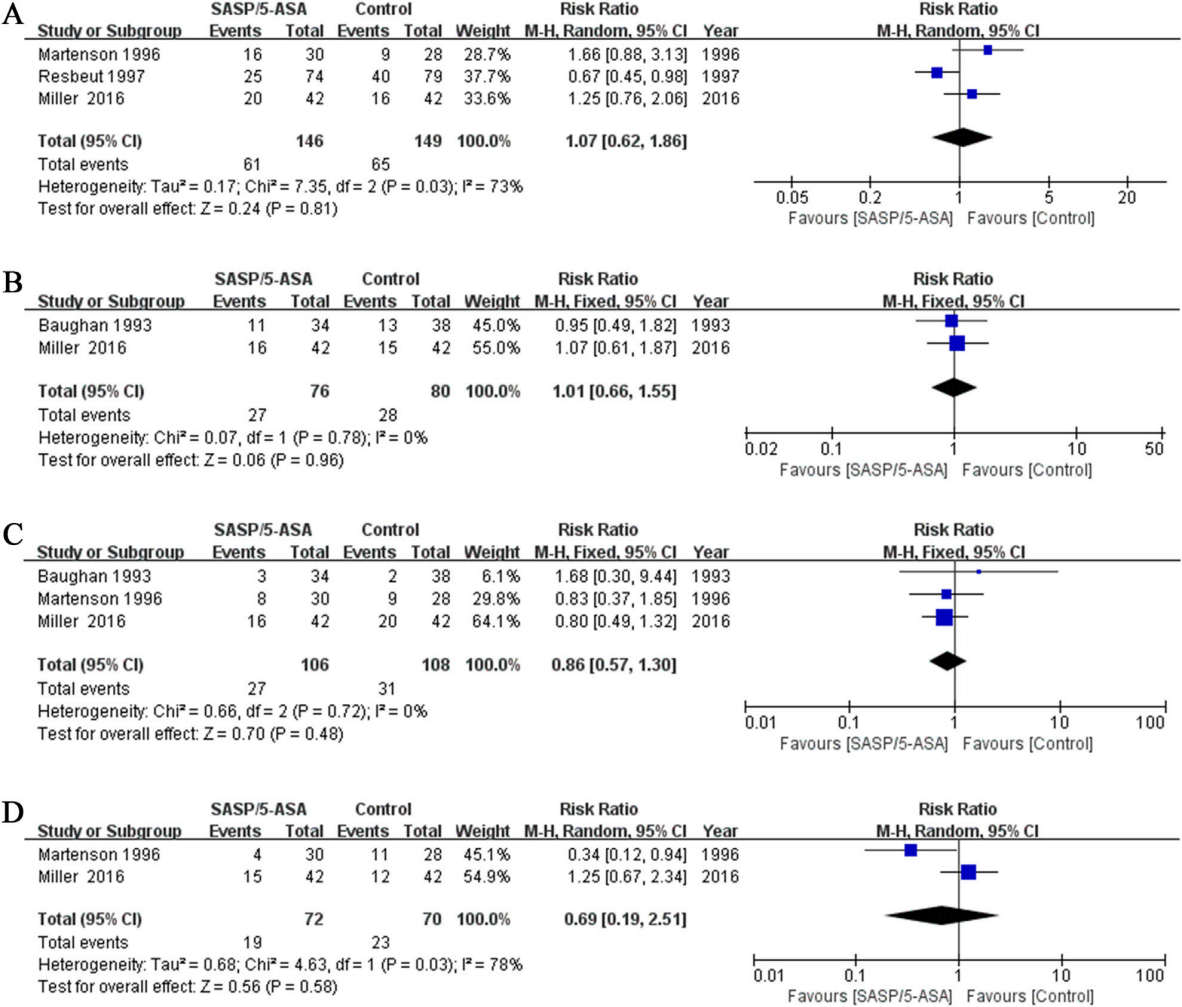
The forest plots of RR for aminosalicylate in relieving different symptoms in patients with ARE. **(A)** Abdominal pain. **(B)** Constipation. **(C)** Hematochezia. **(D)** Tenesmus.

Three studies evaluated the efficacy of aminosalicylates on relieving abdominal pain in patients with ARE. The results showed that the incidence of abdominal pain was similar between the aminosalicylates and the control groups (41.8% vs. 43.6%; Q = 7.35, I^2^ = 73%, P = 0.03; Z = 0.24, P = 0.81; Figure 3A). This suggests that aminosalicylates may not reduce the incidence of abdominal pain.

#### Constipation

Two studies evaluated the incidence of constipation in different groups of patients with ARE. The results showed that 35.5% (27/76) of the patients in the aminosalicylates group, and 35.0% (28/80) in the control group suffered constipation (Q = 0.07, I^2^ = 0.0%, P = 0.78; Z = 0.06, P = 0.96, Figure 3B). This suggests that aminosalicylates may not reduce the incidence of constipation.

#### Hematochezia

Three studies compared the incidence of hematochezia in different groups of patients with ARE. The results indicated that the incidence of hematochezia was similar between the aminosalicylates and the control groups (25.5% vs. 28.7%; Q = 0.66, I^2^ = 0.0%, P = 0.72; Z = 0.70, P = 0.48; Figure 3C). This suggests that aminosalicylates may not reduce the incidence of hematochezia.

#### Tenesmus

Two studies analyzed the incidence of tenesmus in patients with ARE. Although there was a trend toward a decrease in the incidence of tenesmus in the aminosalicylates group, the difference was not statistically significant (26.4% vs. 32.9%; Q = 4.63, I^2^ = 78%, P = 0.03; Z = 0.56, P = 0.58; Figure 3D). This suggests that aminosalicylates may not reduce the incidence of tenesmus.

#### Subgroup analysis

**FIGURE 4:**
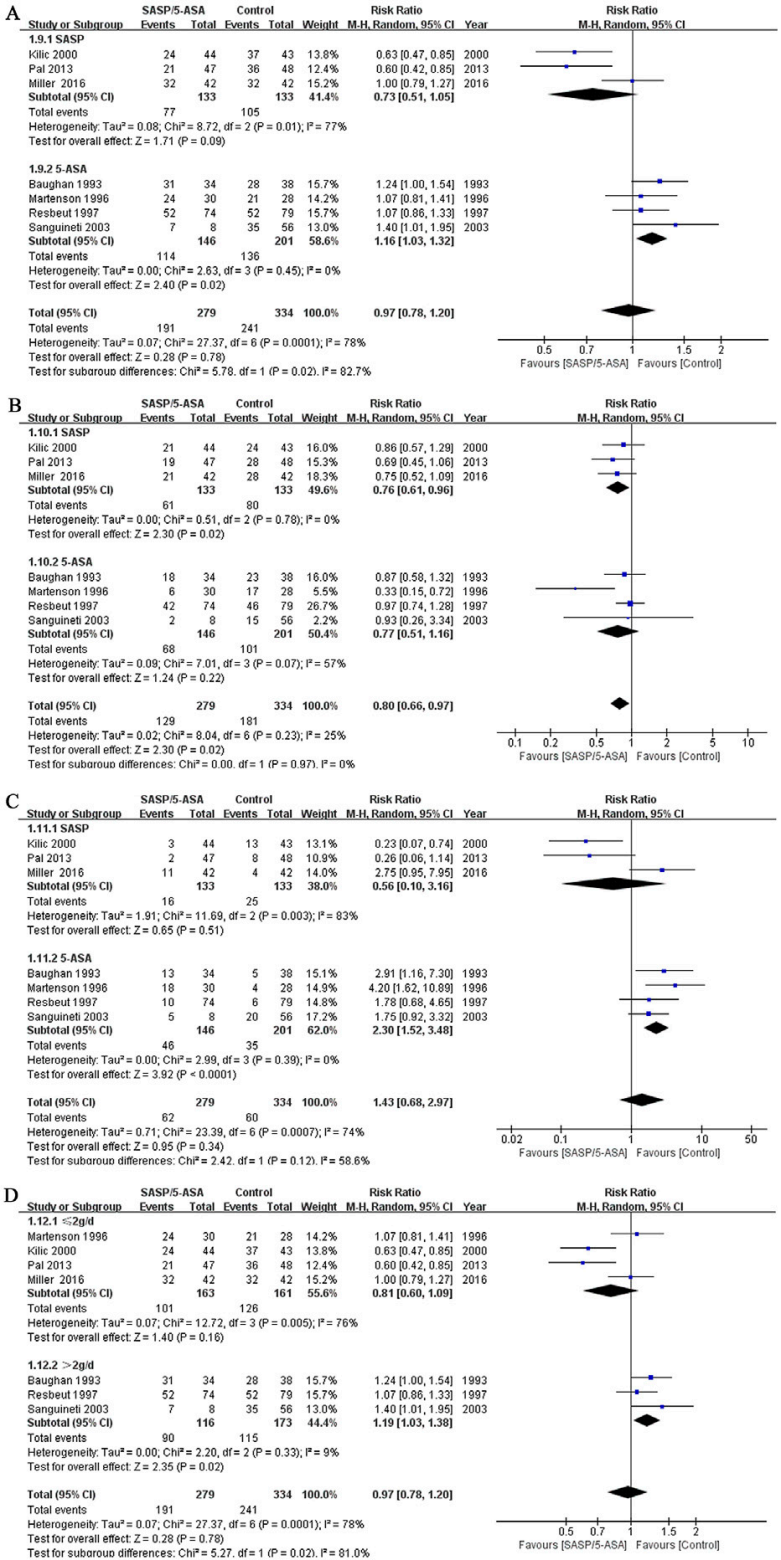
The forest plots of RR for different subgroups of aminosalicylate in relieving diarrhea in patients with ARE. **(A)** Total diarrhea (SASP and 5-ASA subgroups). **(B)** Mild to moderate diarrhea (SASP and 5-ASA subgroups). **(C)** Severe diarrhea (SASP and 5-ASA subgroups). **(D)** Total diarrhea (aminosalicylate ≤2 g/d and aminosalicylate >2 g/d subgroups).

A subgroup analysis based on different types of aminosalicylate was conducted according to the efficacy on alleviating diarrhea. Although the incidence of total diarrhea was lower in the SASP group than that in the control group (57.9% vs. 78.9%; Q = 8.72, I^2^ = 77%, P = 0.01; Z = 1.71, P = 0.09, Figure 4A), the difference was not significant. However, when we analyzed the data of mild to moderate diarrhea among these cases, a lower incidence was identified in the SASP group as compared to the control group (45.9% vs. 60.2%; Q = 0.51, I^2^ = 0.0%, P = 0.78; Z = 2.30, P = 0.02; Figure 4B). Meanwhile, the incidence of severe diarrhea was similar in the SASP group as compared to the control group (12.0% vs. 18.8%; Q = 11.69, I^2^ = 83%, P = 0.003; Z = 0.65, P = 0.51; Figure 4C). These results suggest SASP may be beneficial to the ARE patients with mild to moderate diarrhea but not the severe cases.

The incidence of total diarrhea in the 5-ASA group was higher compared to the control group (78.1% vs. 67.7%; Q = 2.63, I^2^ = 0.0%, P = 0.45; Z = 2.40, P = 0.02; Figure 4A). For the incidence of mild to moderate diarrhea, the results were comparable between the two groups (46.6%vs. 50.2%; Q = 7.01, I^2^ = 57%, P = 0.07; Z = 1.24, P = 0.22; Figure 4B). However, 5-ASA significantly increased the incidence of severe diarrhea in the patients with ARE comparing to the control drugs (31.5% vs. 17.4%; Q = 2.99, I^2^ = 0.0%, P = 0.39; Z = 3.92, P = 0.000; Figure 4C). For this subgroup, 5-ASA did not show superiority, and may even aggravate diarrhea.

Another subgroup analysis based on the dose of aminosalicylates was conducted. Seven studies were included. The incidence of diarrhea was comparable between the group treated with aminosalicylates ≤2 g/d and the control group (62.0% vs. 78.3%; Q = 12.72, I^2^ = 76%, P = 0.005; Z = 1.40, P = 0.16; Figure 4D). However, when the dose of aminosalicylates was escalated to >2 g/d, a higher incidence of diarrhea was reported in the aminosalicylates group comparing to the control group (77.6% vs. 66.5%; Q = 2.20, I^2^ = 9%,P = 0.33; Z = 2.35, P = 0.02, Figure 4D). These results indicated that low dose aminosalicylates may be safe while higher dose may worsen the symptoms of ARE.

### Bias evaluation

**FIGURE 5:**
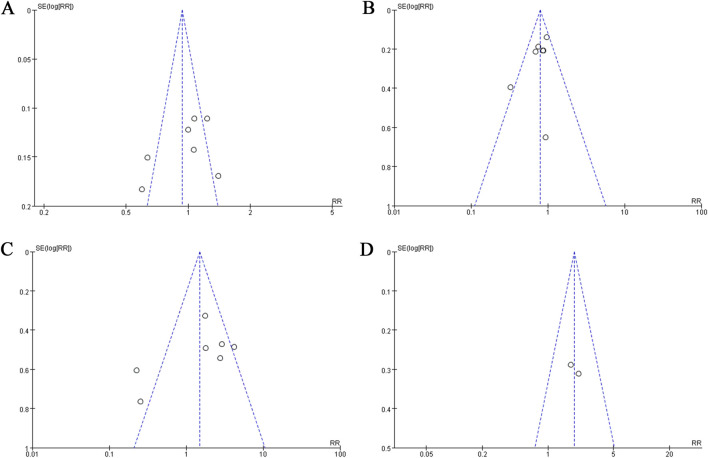
The funnel plots of evaluating publication bias of the studies assessing aminosalicylate in relieving diarrhea in patients with ARE. **(A)** Studies assessing total diarrhea. **(B)** Studies assessing mild to moderate diarrhea. **(C)** Studies assessing severe diarrhea. **(D)** Studies assessing the use of antidiarrheal medications.

**FIGURE 6:**
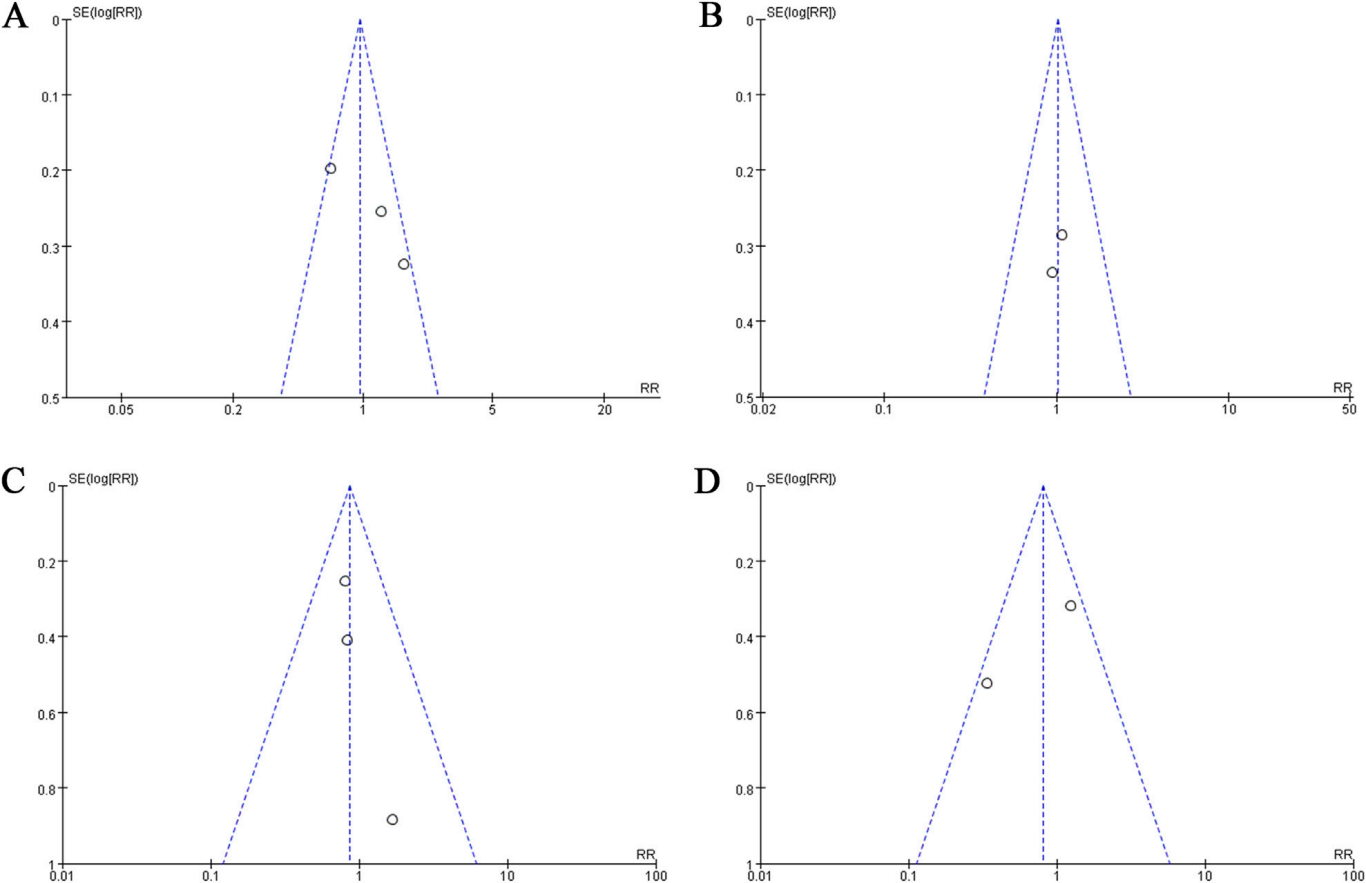
The funnel plots of evaluating publication bias of the studies assessing aminosalicylate in relieving different symptoms in patients with ARE. **(A)** Studies assessing abdominal pain. **(B)** Studies assessing constipation. **(C)** Studies assessing hematochezia. **(D)** Studies assessing tenesmus.

Based on the assessment of publication bias by funnel plot, the RCTs analyzing different symptoms and treatments were basically symmetrical (Figures 5, 6). However, the numbers of RCTs included in each analysis were small, which may lead to publication bias.

### Safety and adverse reactions

Some studies monitored and reported adverse events of aminosalicylates, most of which were vomiting, conjunctivitis, and skin rashes. However, the cause of the vomiting could not be determined since the patients were receiving chemoradiotherapy simultaneously.

## Discussion

Studies had shown that the symptoms associated to ARE are primarily caused by gastrointestinal irritation and inflammation (Martenson et al., 1996). Radiotherapy induces the excessive production of oxygen radicals and inflammatory mediators, such as prostaglandins (PGs), leukotrienes (LTs), and thromboxanes (TXs) (Hubenak et al., 2014; Najafi et al., 2018). Aminosalicylates have been widely used in the treatment of inflammatory bowel disease (IBD) due to their anti-inflammatory and immunomodulatory effects. The pathogenesis of RE and IBD are similar to some extent. Aminosalicylates have been used to treat ARE in recent decades, but the efficacy has been controversial.

We conducted a meta-analysis to evaluate the efficacy of aminosalicylates in treating ARE, incorporating data from seven RCTs. The incidence of overall diarrhea was comparable between the aminosalicylate and control groups (68.5% vs. 72.2%, P > 0.05). However, in the aminosalicylates group, the incidence of mild-to-moderate diarrhea was significantly lower than in the control group (46.2% vs. 54.2%, P < 0.05), whereas the incidence of severe diarrhea remained similar between both groups (22.2% vs. 18.0%, P > 0.05). These findings suggest that while aminosalicylates do not significantly reduce the overall incidence of diarrhea or severe cases, they may be beneficial in alleviating mild-to-moderate diarrhea.

SASP was the first aminosalicylate introduced for IBD treatment (Cooke, 1969). SASP is a compound that includes 5-ASA and sulfapyridine (SP) which are connected by azo bond. The azo bonds in SASP and olsalazine are primarily cleaved by bacterial azo reductases in the ileum and colon, releasing the active component 5-ASA. Later studies found that the adverse effects of SASP were mainly attributable to SP. To mitigate these side effects, newer formulations such as olsalazine and mesalazine were developed, with mesalazine containing only 5-ASA. Olsalazine consists of two 5-ASA molecules linked by an azo bond. Most oral 5-ASA formulations are pH-dependent and release the drug in different sections of the intestinal tract. However, olsalazine and mesalazine have been reported to cause watery diarrhea in some IBD patients. This effect is thought to result from a concentration-dependent inhibition of Na + -K + -ATPase in the ileum and colon, reducing water and electrolyte absorption while increasing secretion, ultimately leading to fluid overload in the colon (Scheurlen et al., 1993). Previous studies also found that osalazine and mesalazine increased the incidence of diarrhea in patients with pelvic radiotherapy (Martenson et al., 1996; Miller et al., 2016). Martenson et al. (1996) proposed that this adverse effect is similarly linked to Na + -K + -ATPase inhibition, which reduces ileal bile salt absorption and induces secretory diarrhea. However, some studies indicated that SASP could reduce the severity of diarrhea occurring in radiation (Kiliç et al., 2000; Pal et al., 2013).

We conducted a subgroup analysis for different pharmaceutical preparations of aminosalicylates. Our results showed that the incidence of overall and severe diarrhea was significantly higher in the 5-ASA group compared to the control group (78.1% vs. 67.7%, 31.5% vs. 17.4%, P < 0.05), while the SASP group exhibited lower diarrhea rates than the control group, though the difference was not statistically significant. Interestingly, SASP significantly reduced the incidence of mild-to-moderate diarrhea compared to the control group (45.9% vs. 60.2%, P < 0.05).

Previous studies have found that taking drugs containing prostaglandin may cause diarrhea (Barr and Naismith, 1972). Mennie et al. (1975) speculated that prostaglandin release might be a contributing factor in radiation-induced diarrhea. However, the differing effects of 5-ASA and SASP have led some researchers to question the precise role of eicosanoids in the pathogenesis of ARE. This has given rise to the hypothesis that 5-ASA and SASP may act through distinct mechanisms (Sanguineti et al., 2003). The failure of 5-ASA in treating ARE suggests that the sulfonamide component of SASP may be responsible for its diarrhea-reducing effects in ARE (Sanguineti et al., 2003).

Chemotactic peptide N-Formyl-L-methionyl-L-leucyl-L-phenylalanine (FMLP) is a bacteria-derived peptide that binds to a specific receptor on neutrophil, monocyte and macrophage, which leads to neutrophil chemotaxis and superoxide production (Cavicchioni et al., 2006). SASP has been shown to dose-dependently inhibit FMLP binding to neutrophils and suppress FMLP-induced inflammation and chemotaxis, whereas 5-ASA exhibits a weaker effect (Stenson et al., 1984). The metabolites of LOX, cysteinyl leukotrienes (LTC4 and LTD4), can strongly increase the smooth muscle contractility and enhance mucus secretion and vascular permeability (Singh et al., 2010; Sasaki and Yokomizo, 2019). While SASP effectively inhibits the formation of these metabolites, 5-ASA exerts only a minimal effect (Nielsen et al., 1988).

Some studies found that the level of Thromboxane B2 (TXB2) was reduced and the colitis would be improved when the specific inhibitor to thrombin synthase, OKY1581, was given to the IBD patients (Vilaseca et al., 1990). Hawkey et al. (1985) found that SASP inhibited the production of TXB in the intestinal tissue homogenate induced by arachidonic acid, whereas 5-ASA has no such effect. Whether the above mentioned mechanisms can explain the differences between 5-ASA and SASP in treating ARE needs further studies to validate.

Currently, no established consensus exists regarding the optimal dosage of aminosalicylates for ARE treatment. Previous studies (Kruis et al., 1998; Sann et al., 2013) indicate that different formulations may have varying effects on diarrhea. The aminosalicylate doses in our study ranged from 1 g/day to 4 g/day. Our analysis revealed that the incidence of diarrhea was significantly higher in patients receiving aminosalicylates at doses >2 g/day compared to the control group (77.6% vs. 66.5%, P < 0.05). Prior research suggests that the watery diarrhea caused by olsalazine and mesalazine in IBD patients is dose-dependent (Scheurlen et al., 1993). Our findings align with these studies, indicating that using aminosalicylates at doses ≤2 g/day may be relatively safe in ARE.

Furthermore, our study found that aminosalicylates did not demonstrate superiority over control treatments in alleviating ARE symptoms such as abdominal pain, constipation, hematochezia, and tenesmus. Few adverse events were reported in these RCTs, and no severe adverse events were identified, suggesting that aminosalicylates are generally well-tolerated and safe. However, further evidence is needed due to variations in primary diseases, concurrent chemotherapy regimens, radiation doses, small sample sizes, and study heterogeneity within this meta-analysis.

A limitation of the included studies is that they relied solely on clinical symptoms to assess the severity of ARE. Incorporating endoscopy as an additional evaluation method would provide more objective evidence regarding the efficacy of aminosalicylates. Moreover, the use of antidiarrheal medications in some studies may have confounded the assessment of aminosalicylates’ therapeutic effects in ARE. Additionally, none of the included studies evaluated or reported the prevalence of opportunistic infections, which could be an important consideration given the potential impact of immunosuppression or gut microbiota alterations in patients receiving aminosalicylates. Further randomized controlled trials are needed to validate their efficacy and assess the potential role of opportunistic infections in treatment outcomes.

## Conclusion

In summary, SASP appears to be effective in preventing and treating mild to moderate diarrhea in ARE, while 5-ASA may be associated with an increased incidence of total and severe diarrhea in patients undergoing pelvic radiotherapy. A dosage of aminosalicylates ≤2 g/day is relatively safe for the treatment of ARE, whereas higher doses may worsen diarrhea. Given the limitations of this study, future well-designed randomized controlled trials with standardized dosing regimens and objective outcome measures are needed to further confirm the efficacy and safety of aminosalicylates in ARE.

## Data Availability

The original contributions presented in the study are included in the article/supplementary material, further inquiries can be directed to the corresponding authors.
